# Buccal dantrolene for rapid systemic delivery: A novel antiarrhythmic approach

**DOI:** 10.1016/j.hroo.2025.11.012

**Published:** 2025-11-25

**Authors:** Sofiane Ouerd, Praloy Chakraborty, Stéphane Massé, Patrick F.H. Lai, Eric X. Chen, Eitan Amir, Nigel deGruyther, Kumaraswamy Nanthakumar

**Affiliations:** 1 Toronto General Hospital, University Health Network, Toronto, Ontario, Canada; 2 Princess Margaret Cancer Centre, University Health Network, Toronto, Ontario, Canada; 3 University of Toronto, Toronto, Ontario, Canada

**Keywords:** Dantrolene, Antiarrhythmic drugs, Cardiac ryanodine channels, Calcium signaling, Ventricular arrhythmias

**Key Findings**

▪ A novel mucoadhesive, water-soluble buccal film formulation of dantrolene was developed to enable systemic absorption while bypassing hepatic first-pass metabolism.
▪ Buccal administration of dantrolene in the porcine model resulted in early detectability and a time-dependent increase in serum concentrations.
▪ Serum concentrations varied with formulation pH, with significantly higher levels achieved using more alkaline films.
▪ Dantrolene was detectable in serum as early as 10 minutes after buccal application.
▪ No local adverse effects, such as hyperemia, bleeding, or ulceration, were observed on the buccal mucosa.

Pharmacologic control of ventricular arrhythmia remains challenging, especially in the presence of structural heart disease, a condition characterized by borderline hemodynamics and extensive cardiac structural and electrophysiological remodeling. Conventional antiarrhythmic agents may exacerbate arrhythmias by prolonging action potential duration and/or repolarization heterogeneity (class III agents) or slowing ventricular conduction (class IA agents).^1^ Simultaneously, the negative inotropic or vasodilatory effects of current antiarrhythmic agents may worsen hemodynamic instability.^1^

Cardiac ryanodine receptor 2 channel modulation by dantrolene and its water-soluble analog azumolene has been shown to reduce arrhythmogenic calcium dysregulation and arrhythmia without causing proarrhythmic action potential duration prolongation, action potential alternans, or slowing of phase 1 depolarization.^2^^,^^3^ In addition, ryanodine receptor 2 modulation did not impair contractility in end-stage heart failure.^4^ These findings make dantrolene a promising antiarrhythmic candidate.

Dantrolene is currently available as capsules and intravenous formulations. Oral (enteral) dantrolene is characterized by slow and variable absorption, delaying its onset, a significant drawback in acute cardiac emergencies. Although intravenous dantrolene offers a faster onset, it is limited by poor solubility and requires high-volume infusion, making it impractical for administration outside hospital settings. To overcome these barriers of poor pharmacokinetics, we developed a buccal film formulation of dantrolene, designed for mucosal absorption in the oral cavity and bypassing hepatic metabolism. In collaboration with Nova Thin Film Pharmaceuticals, a company specializing in noninvasive drug delivery, we used their DepoFilm technology to produce a novel mucoadhesive, water-soluble film that enables systemic uptake via the buccal mucosa. Using a porcine model, we investigated the feasibility of achieving therapeutic serum concentrations via buccal delivery and whether drug uptake is influenced by the pH of the film formulation. The research reported in this paper adhered to the Animal Research: Reporting of In Vivo Experiments and Guide for the Care and Use of Laboratory Animals guidelines. The study was approved by the Institutional Animal Care and Use Committee. 13 anesthetized male Yorkshire pigs (body weight 24.7 ± 2.2 kg) were randomly assigned to receive dantrolene buccal films (dose 4 ± 0.4 mg/kg) formulated at pH 4 (n = 3), 7.5 (n = 3), 9.0 (n = 4), or 10.4 (n = 3). Blood samples were collected percutaneously from a femoral or radial artery for serum dantrolene estimation at baseline (immediately before film placement on the buccal surface), 10 minutes, 30 minutes, and then every 30 minutes up to 240 minutes. A high-performance liquid chromatography–tandem mass spectrometry–based assay was performed to determine serum dantrolene levels. Dantrolene was detectable in serum by 10 minutes with a gradual, linear increase over time (*P* < .0001) (Figure 1A). At each time point, serum dantrolene levels differed significantly by pH (*P* = .0376) and were consistently higher with more alkaline formulations. Serum dantrolene concentration at 240 minutes was influenced significantly by pH (*P* = .0019), with the highest levels observed at pH 10.4, followed by pH 9, compared with pH 4 and pH 7.5 (Figure 1B). No signs of hyperemia, bleeding, or ulceration were observed on the buccal mucosa.

**Figure 1:**
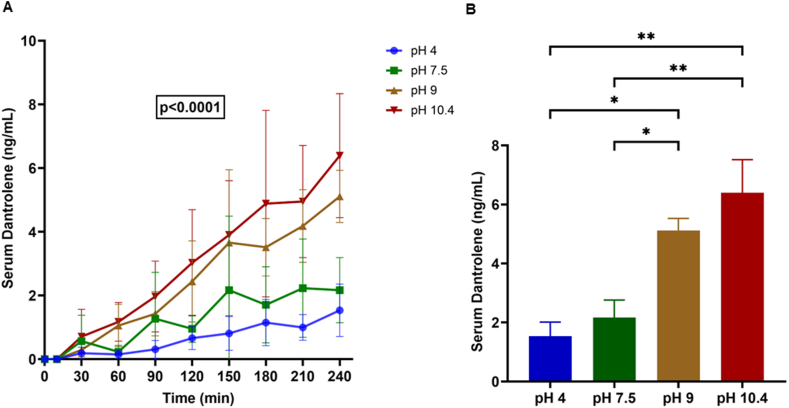
Serum dantrolene levels after buccal film administration. Serum concentrations increased in a time- and pH-dependent manner (panel A, 2-way analysis of variance). Serum levels at 240 minutes were significantly influenced by formulation pH (panel B, 1-way analysis of variance). ∗*P* < .05 and ∗∗*P* < .01 vs respective control.

Although this study established the feasibility of dantrolene delivery using the buccal film technology, serum concentrations (maximum serum levels after 4 hours 1.5–6.4 ng/mL) were not as high as those reported after a comparable enteral dose (5 mg/kg) in a canine model (maximum concentration 0.43 μg/mL).^5^ This discrepancy likely reflects limited mucosal absorption, formulation constraints (eg, solubility at lower pH), a shorter sampling window, and species-related pharmacokinetic differences. Nevertheless, dantrolene was detectable by 10 minutes, and serum levels rose in a time- and pH-dependent manner, supporting the potential of buccal delivery and the importance of pH of the film formulation in drug delivery. These findings provide a foundation for further optimization of film composition and dosing. Buccal administration may offer a viable parenteral alternative to existing formulations of dantrolene, particularly for emergency arrhythmia management in out-of-hospital settings.

## Disclosures

K.N. holds a patent on dantrolene for antiarrhythmic therapy. No other authors have any conflicts of interest to disclose.
